# Baseline facial emotion recognition is associated with clinical decline across the Alzheimer’s disease spectrum: a multi-instrument longitudinal assessment study

**DOI:** 10.3389/fnagi.2026.1863175

**Published:** 2026-06-30

**Authors:** YongSoo Shim, Eun Ye Lim

**Affiliations:** 1 Department of Neurology, St. Vincent’s Hospital, College of Medicine, The Catholic University of Korea, Seoul, Republic of Korea; 2 Department of Neurology, Eunpyeong St. Mary’s Hospital, College of Medicine, The Catholic University of Korea, Seoul, Republic of Korea

**Keywords:** Alzheimer’s disease, CDR-SOB, facial emotion recognition, longitudinal, mild cognitive impairment, mixed-effects model, MMSE, social cognition

## Abstract

**Background:**

Facial emotion recognition (FER) is increasingly recognized as a functionally relevant domain in the Alzheimer’s disease (AD) spectrum, yet whether baseline FER is associated with subsequent clinical decline remains insufficiently established.

**Methods:**

We studied 328 participants [subjective cognitive decline (SCD) *n* = 94, mild cognitive impairment (MCI) *n* = 142, AD dementia *n* = 92] from a longitudinal memory clinic cohort; 148 had follow-up assessments (mean 1.59 ± 0.67 years). FER was assessed using the Korean version of the Florida Affect Battery (K-FAB; five subtests, FER_mean). Linear mixed-effects models examined group-differential trajectories of the Mini-Mental State Examination (MMSE), Clinical Dementia Rating–Sum of Boxes (CDR-SOB), and FER_mean, adjusted for age, sex, education, and baseline values.

**Results:**

FER_mean decreased stepwise across groups at baseline (SCD 17.3 ± 1.5, MCI 16.4 ± 2.1, AD 15.3 ± 2.0; *p* < 0.001). CDR-SOB showed more consistent group-differential longitudinal trajectories: MCI increased at +0.550 points/year (*p* < 0.001), with SCD significantly slower (*β* = −0.491, *p* = 0.008) and AD faster (*β* = +0.371, *p* = 0.038). FER_mean showed a modest decline in MCI (*β* = −0.169/yr., *p* = 0.078) and SCD showed significantly attenuated decline relative to MCI (*β* = +0.376, *p* = 0.035). Lower baseline FER was associated with faster CDR-SOB worsening (*ρ* = −0.221, *p* = 0.006) but not MMSE decline (*ρ* = 0.093, *p* = 0.255). FER change rates showed a weak, non-significant correlation with MMSE change (*ρ* = 0.004, *p* = 0.960) but a modest significant correlation with CDR-SOB change (*ρ* = −0.195, *p* = 0.018), suggesting partially distinct longitudinal patterns.

**Conclusion:**

Baseline FER was modestly associated with longitudinal worsening in clinical severity (CDR-SOB), but not cognitive screening performance (MMSE) across the pre-dementia to dementia spectrum. FER trajectories showed limited correlation with traditional measures. These findings indicate that FER may have a potential complementary role of social cognitive assessment in the AD continuum, pending further validation.

## 1 Introduction

Alzheimer’s disease (AD) is a heterogeneous clinical syndrome that extends well beyond episodic memory impairment. In recent years, social cognition—encompassing the ability to perceive, interpret, and respond to social and emotional cues—has emerged as a functionally relevant but underappreciated domain within the AD spectrum (Černotová et al., 2023; Cosentino et al., 2014; Shi et al., 2025; Shim et al., 2025). Among social cognitive processes, facial emotion recognition (FER) plays a critical role in everyday interpersonal communication and has been shown to be impaired in patients with established AD dementia (Burnham and Hogervorst, 2004; Hargrave et al., 2002; Kohler et al., 2005).

Cross-sectional evidence suggests that FER deficits may emerge early in the disease continuum, including in subjective cognitive decline (SCD) and mild cognitive impairment (MCI) (Pietschnig et al., 2016; Shim, 2023; Teng et al., 2007). SCD represents the earliest symptomatic stage, characterized by self-perceived cognitive difficulties without objective impairment, whereas MCI reflects objectively measurable decline with preserved functional independence. Both stages confer elevated risk for subsequent dementia (Ávila-Villanueva and Fernández-Blázquez, 2017; Eckerström et al., 2017; Hong and Lee, 2017; Jessen et al., 2014; Jessen and Rodriguez, 2018; Kang et al., 2024; van Harten et al., 2018), underscoring the clinical importance of identifying early prognostic markers.

Despite growing cross-sectional evidence, several critical gaps remain. First, longitudinal data on FER trajectories across normal cognition/SCD/MCI/dementia groups are scarce, with most prior studies relying on single time-point assessments (Shim, 2023). Second, comparative studies examining the rate of FER change alongside established cognitive and clinical outcome measures—particularly the Mini-Mental State Examination (MMSE) and the Clinical Dementia Rating–Sum of Boxes (CDR-SOB)—are nearly absent. Third, it remains unclear which of these clinical instruments more closely tracks longitudinal FER change, despite their differing sensitivity to disease severity (Benoit et al., 2020). Fourth, whether baseline FER performance independently predicts subsequent cognitive or clinical worsening in pre-dementia stages remains insufficiently established.

In this longitudinal cohort study, we addressed these gaps by: (1) characterizing FER and cognitive trajectories across SCD, MCI, and AD groups using linear mixed-effects models; (2) comparing annual rates of change across instruments and diagnostic groups; (3) examining the interrelationships among longitudinal changes in multiple clinical assessment instruments; and (4) evaluating whether baseline FER is associated with subsequent changes in MMSE and CDR-SOB. We hypothesized that baseline FER performance would be associated with subsequent clinical decline, as indexed by CDR-SOB and MMSE changes.

## 2 Materials and methods

### 2.1 Participants

Participants were drawn from an ongoing longitudinal cohort at a university-affiliated memory clinic. Inclusion criteria were: (1) baseline diagnosis of SCD, MCI, or AD dementia; (2) availability of baseline FER assessment; and (3) availability of concurrent MMSE, CDR, and CDR-SOB assessments. Patients with a CDR global score exceeding 1 were excluded. For longitudinal analyses, at least one follow-up visit with repeated FER, MMSE, CDR, and CDR-SOB measurements was required. Baseline diagnosis was established by consensus of experienced neurologists based on standardized neuropsychological evaluation, clinical history, and neuroimaging. SCD was diagnosed according to the criteria proposed by Jessen et al. (2014); MCI was defined according to Petersen criteria (Petersen, 2004) and the NIA-AA guidelines (Albert et al., 2011); AD dementia was diagnosed using NIA-AA criteria (McKhann et al., 2011).

In total, 328 participants with valid baseline data were included in cross-sectional analyses (SCD *n* = 94, MCI *n* = 142, AD *n* = 92). Of these, 148 participants (SCD *n* = 30, MCI *n* = 67, AD *n* = 51) had at least one follow-up FER, MMSE, CDR, and CDR-SOB assessment and comprised the longitudinal cohort. The Institutional Review Board of the Catholic University of Korea, Eunpyeong St. Mary’s Hospital approved the study (PC23RISI0170), and informed consent was obtained from all participants or their caregivers. This study was conducted in accordance with the Declaration of Helsinki.

### 2.2 Facial emotion recognition assessment

To evaluate facial emotion recognition ability, we administered the Korean version of the Florida Affect Battery (K-FAB) (Ahn et al., 2009; Shim, 2023), a standardized and validated instrument focusing exclusively on facial affect tasks. The K-FAB consists of five subtests: subtest 1 (facial identity discrimination: participants judge whether two sequentially presented faces belong to the same or a different person, independent of emotional expression); subtest 2 (facial affect discrimination: participants judge whether two faces display the same or a different emotional expression); subtest 3 (facial affect naming: participants verbally identify the emotion displayed by a face, selecting from five categories—happiness, sadness, anger, fear, and neutrality); subtest 4 (facial affect selection: participants select, from an array of five faces, the one that matches a verbally provided emotion label); and subtest 5 (facial affect matching: participants select, from an array of five faces, the one displaying the same emotion as a target face). Each subtest presents 20 facial stimuli consisting of static, standardized black-and-white photographs of facial expressions from non-actor individuals. Overall FER performance was summarized as the mean score across all five subtests (FER_mean; maximum 20 per subtest). The date of the FER assessment served as the baseline time point for longitudinal analyses, and follow-up time (years) was calculated from this reference date.

### 2.3 Cognitive and clinical outcome measures

Longitudinal outcomes included: (1) the Mini-Mental State Examination (MMSE; range 0–30; higher scores indicate better performance) (Baek et al., 2016; Folstein et al., 1975); (2) the Clinical Dementia Rating Scale Sum of Boxes (CDR-SOB; range 0–18; higher scores indicate greater impairment) (Morris et al., 1996); and (3) FER_mean. Baseline outcome values were included as covariates in all longitudinal models to adjust for initial disease severity.

### 2.4 Statistical analysis

Baseline group differences were examined using one-way ANOVA for continuous variables and chi-square tests for categorical variables. Post-hoc pairwise comparisons were conducted using Tukey’s HSD correction.

Individual-level annual rates of change (slopes) were estimated for each participant using simple linear regression of each outcome on time (years). Group-level differences in slopes were assessed by one-way ANOVA.

Linear mixed-effects models (LMMs) with random intercepts for participants examined group-differential trajectories. The primary model was specified as:


$$\begin{array}{c} \text{Outcome}\_\mathrm{it}=\beta 0+\beta 1\cdot \text{Time}\_\mathrm{it}+\beta 2\cdot \text{Group}\_\mathrm{SCD}\_i+\\ \beta 3\cdot \text{Group}\_\mathrm{AD}\_i.+\\ \beta 4\cdot (\text{Time}\_\mathrm{it}\times \text{Group}\_\mathrm{SCD}\_i)+\\ \beta 5\cdot (\text{Time}\_\mathrm{it}\times \text{Group}\_\mathrm{AD}\_i)+\\ \beta 6\cdot \mathrm{Age}\_i+\beta 7\cdot \mathrm{Sex}\_i+\\ \beta 8\cdot \text{Education}\_i+\beta 9\cdot \text{Baseline}\_\\ \text{Outcome}\_i+(1\;|\;\text{Subject}\_i) \end{array}$$

MCI served as the reference group. The interaction terms (Time × Group) represent differential rates of change relative to MCI. A significant positive interaction with Group_SCD indicates slower worsening in SCD versus MCI; a significant positive interaction with Group_AD for CDR-SOB indicates faster worsening in AD.

Associations between baseline FER and individual rates of change in MMSE and CDR-SOB were assessed using Spearman’s rank correlation (*ρ*) across the combined FU cohort (SCD + MCI + AD). Correlations between change rates of MMSE, CDR-SOB, and FER were likewise computed using Spearman’s rank correlation to examine whether instruments capture overlapping or distinct aspects of disease progression. Shapiro–Wilk tests confirmed significant deviation from normality for all annual slope distributions (all *p* < 0.001), justifying the use of non-parametric correlation.

All analyses were performed in Python (version 3.14) using statsmodels, scipy, and scikit-learn libraries. Statistical significance was set at *α* = 0.05 (two-tailed).

## 3 Results

### 3.1 Baseline characteristics

Baseline characteristics are summarized in Table 1. The cross-sectional cohort included 94 SCD, 142 MCI, and 92 AD participants. Groups did not differ significantly in sex distribution (female: SCD 72%, MCI 70%, AD 64%; *p* = 0.463), years of education (SCD 9.6 ± 4.3, MCI 9.3 ± 4.9, AD 9.3 ± 4.9 years; *p* = 0.889), or age (SCD 73.4 ± 7.8, MCI 72.6 ± 17.6, AD 77.3 ± 16.0 years; *p* = 0.054). All cognitive and clinical measures differed significantly across groups (all *p* < 0.001). MMSE scores decreased stepwise (SCD 27.2 ± 2.5, MCI 24.9 ± 3.5, AD 20.0 ± 4.3), CDR-SOB increased (SCD 1.14 ± 0.74, MCI 1.60 ± 0.97, AD 5.35 ± 2.24), and FER_mean decreased (SCD 17.3 ± 1.5, MCI 16.4 ± 2.1, AD 15.3 ± 2.0; all *p* < 0.001).

**Table 1:** Baseline characteristics by diagnostic group (mean ± SD unless stated).

Variable	SCD (*n* = 94)	MCI (*n* = 142)	AD (*n* = 92)	*p* (ANOVA/χ^2^)
FU available, *n* (%)	30 (32%)	67 (47%)	51 (55%)	—
Female, *n* (%)	68 (72%)	99 (70%)	59 (64%)	0.463
Age (years)	73.4 ± 7.8	72.6 ± 17.6	77.3 ± 16.0	0.054
Education (years)	9.6 ± 4.3	9.3 ± 4.9	9.3 ± 4.9	0.889
MMSE	27.2 ± 2.5	24.9 ± 3.5	20.0 ± 4.3	<0.001
CDR-SOB	1.14 ± 0.74	1.60 ± 0.97	5.35 ± 2.24	<0.001
FER Mean (FAB1–5)	17.3 ± 1.5	16.4 ± 2.1	15.3 ± 2.0	<0.001
FAB1	18.8 ± 1.5	18.4 ± 1.9	17.5 ± 2.2	<0.001
FAB2	17.5 ± 1.4	17.1 ± 1.9	16.5 ± 2.3	<0.001
FAB3	16.8 ± 2.3	15.6 ± 2.9	14.5 ± 2.5	<0.001
FAB4	17.7 ± 2.3	16.8 ± 2.9	15.9 ± 2.5	<0.001
FAB5	15.6 ± 3.3	13.3 ± 4.4	11.7 ± 4.4	<0.001

SCD, subjective cognitive decline; MCI, mild cognitive impairment; AD, Alzheimer’s disease dementia; MMSE, Mini-Mental State Examination; CDR-SOB, Clinical Dementia Rating–Sum of Boxes; FER, facial emotion recognition.

### 3.2 FER subtest profile across groups

All five FER subtests showed significant group differences at baseline (all p < 0.001). FAB5 demonstrated the largest absolute between-group difference (SCD 15.6 ± 3.3, MCI 13.3 ± 4.4, AD 11.7 ± 4.4), followed by FAB3 (SCD 16.8 ± 2.3, MCI 15.6 ± 2.9, AD 14.5 ± 2.5). In contrast, FAB1 showed the smallest between-group separation (SCD 18.8 ± 1.5, MCI 18.4 ± 1.9, AD 17.5 ± 2.2), suggesting a ceiling effect for this subtest. These findings indicate that not all FER subtests are equally sensitive to cognitive severity.

### 3.3 Longitudinal cohort characteristics

Of the 148 participants with follow-up data, mean follow-up duration was 1.59 ± 0.67 years (range 0.59–3.32). Follow-up duration differed significantly across groups (SCD 1.67 ± 0.69 yr., MCI 1.73 ± 0.72 yr., AD 1.37 ± 0.50 yr.; one-way ANOVA: *p* = 0.011), with the AD group showing a shorter mean follow-up period compared with the SCD and MCI groups.

### 3.4 Group-differential longitudinal trajectories (LMM results)

**Figure 1:**
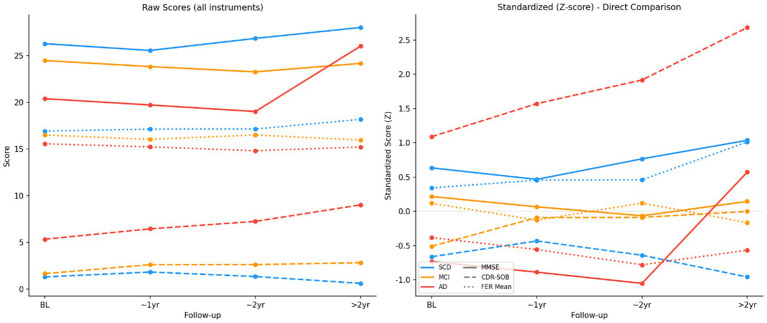
Estimated longitudinal trajectories—multi-instrument comparison. Left: raw score trajectories for all three instruments across groups. Right: standardized (*Z*-score) trajectories enabling direct cross-instrument comparison. AD CDR-SOB demonstrates the most pronounced deviation from baseline after 2 years.

Results of linear mixed-effects models examining group-differential rates of change are illustrated in Figure 1, Supplementary Figures 1, 2.

For CDR-SOB, the MCI reference group showed a significant increase of +0.550 points/year (95% CI: 0.355–0.744; *p* < 0.001). SCD showed a significantly attenuated rate of CDR-SOB increase relative to MCI (*β* = −0.491, 95% CI: −0.852 to −0.130; *p* = 0.008), while AD showed a significantly accelerated rate (*β* = +0.371, 95% CI: 0.021–0.721; *p* = 0.038), corresponding to a total CDR-SOB increase of approximately +0.921 points/year in AD.

For MMSE, the MCI reference group declined at −0.533 points/year (95% CI: −0.901 to −0.164; *p* = 0.005). SCD showed significantly slower MMSE decline than MCI (*β* = +0.721, 95% CI: 0.037–1.405; *p* = 0.039). The AD–MCI difference was not statistically significant (*β* = +0.334, *p* = 0.326), suggesting that after adjusting for baseline disease severity, AD and MCI exhibit comparable MMSE decline trajectories.

For FER_mean, MCI showed a marginal decline of −0.169 points/year (95% CI: −0.356 to 0.019; *p* = 0.078). SCD showed a significantly attenuated FER decline relative to MCI (*β* = +0.376, 95% CI: 0.027–0.725; *p* = 0.035). The AD–MCI difference was negligible and non-significant (*β* = −0.030, *p* = 0.860), indicating that once cognitive severity is controlled, MCI and AD exhibit similar FER deterioration rates.

### 3.5 Annual rate of change comparison

**Figure 2:**
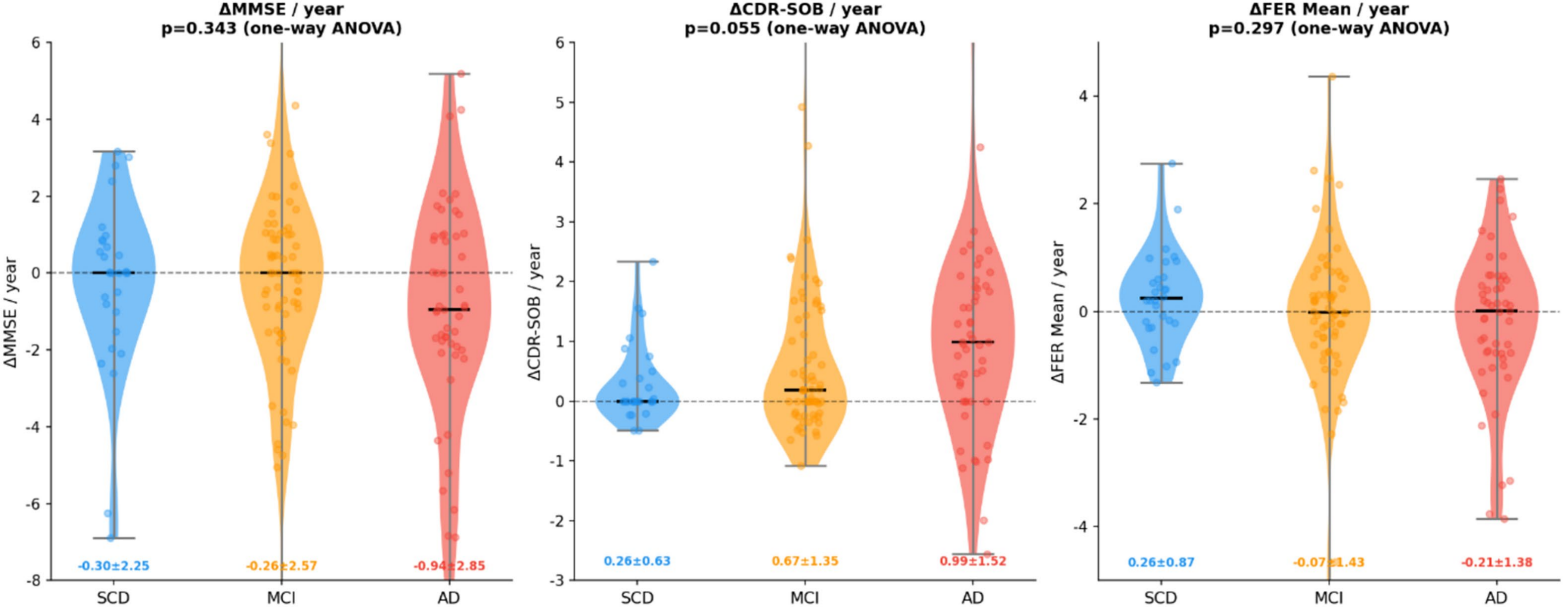
Annual rate of change by group. Violin plots with individual data points showing distributions of annual change rates (*Δ*/year) for MMSE (left), CDR-SOB (middle), and FER mean (right) across SCD, MCI, and AD. Dashed horizontal line at zero. Mean ± SD values annotated below each group. *p* values from one-way ANOVA.

Individual-level annual rates of change are displayed in Figure 2. CDR-SOB change rates showed a monotonic increase across groups (SCD: +0.26 ± 0.63, MCI: +0.67 ± 1.35, AD: +0.99 ± 1.52 points/year; ANOVA *p* = 0.055), approaching statistical significance. MMSE change rates did not differ significantly across groups (SCD: −0.30 ± 2.25, MCI: −0.26 ± 2.57, AD: −0.94 ± 2.85 points/year; *p* = 0.343), consistent with the known ceiling and floor effects of MMSE in the pre-dementia and mild dementia stages. FER_mean change rates were directionally consistent with greater decline in more impaired groups (SCD: +0.26 ± 0.87, MCI: −0.07 ± 1.43, AD: −0.21 ± 1.38 per year; *p* = 0.297) but did not reach significance at the group level, likely reflecting high intra-group variability.

### 3.6 Multi-instrument simultaneous follow-up

To address whether MMSE, CDR-SOB, and FER_mean capture overlapping or distinct aspects of disease progression, we examined correlations between individual annual change rates. MMSE and CDR-SOB change rates were significantly correlated (*ρ* = −0.356, *p* < 0.001), consistent with their measuring related dimensions of cognitive-clinical severity. In contrast, FER_mean change rate showed only a weak, non-significant correlation with MMSE change (*ρ* = 0.004, *p* = 0.960); however, a modest but statistically significant correlation was observed between FER_mean change rate and CDR-SOB change (*ρ* = −0.195, *p* = 0.018). These findings suggest that FER trajectories are largely independent of MMSE-indexed global cognitive change but share modest overlap with CDR-SOB–indexed functional decline.

Standardized (*Z*-score) trajectory plots (Figure 1, right panel) revealed that CDR-SOB provided the most sensitive differentiation of group trajectories over time, with AD showing pronounced worsening beyond the two-year mark relative to MCI and SCD. FER_mean trajectories showed more gradual and overlapping changes across groups, particularly between MCI and AD.

### 3.7 Baseline FER as predictor of longitudinal decline

**Figure 3:**
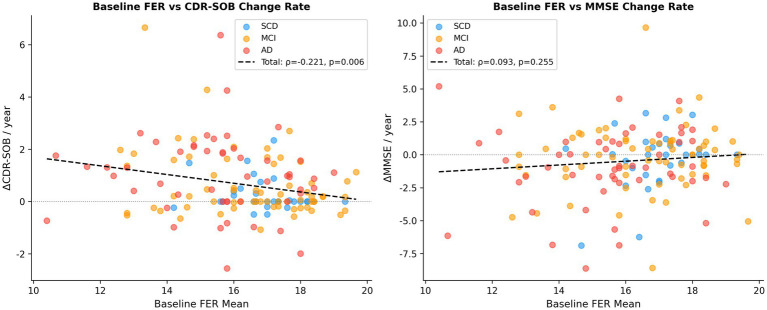
Baseline FER and longitudinal change rate. Scatter plots of baseline FER mean versus annual rate of CDR-SOB change (left) and MMSE change (right) in the follow-up cohort. Dashed lines represent total-sample linear regression fits. *ρ* and *p* values reflect Spearman’s rank correlation across the combined cohort.

Lower baseline FER_mean was associated with a faster rate of CDR-SOB increase over time across the combined FU cohort (*ρ* = −0.221, *p* = 0.006, *n* = 152; Figure 3, left). In contrast, the association between baseline FER and MMSE change rate was weak and not statistically significant (*ρ* = 0.093, *p* = 0.255; Figure 3, right). This dissociation between CDR-SOB and MMSE is consistent with the primary longitudinal analysis and supports the hypothesis that FER reflects vulnerability in functionally relevant domains captured by CDR-SOB rather than global cognitive screening performance.

## 4 Discussion

In this longitudinal cohort study spanning SCD, MCI, and AD dementia, we examined FER trajectories alongside established cognitive and clinical measures to address key gaps in the literature. The principal findings were: (1) FER_mean decreases stepwise across the diagnostic spectrum at baseline; (2) CDR-SOB showed more consistent group-differential longitudinal change than MMSE in mixed-effects models; (3) FER trajectories were largely independent of MMSE change (*ρ* = 0.004, *p* = 0.960) but modestly correlated with CDR-SOB change (*ρ* = −0.195, *p* = 0.018), suggesting partially overlapping functional decline patterns; and (4) lower baseline FER was modestly associated with faster worsening in CDR-SOB—but not decline in MMSE—across the clinically defined pre-dementia to dementia spectrum.

The cross-sectional FER gradient across SCD → MCI → AD is consistent with prior evidence that social cognitive deficits emerge early in the AD spectrum (Shim, 2023). Among FER subtests, FAB5 showed the largest between-group difference, suggesting that certain emotional categories or task demands may be particularly sensitive to early neural degeneration in AD-related networks (Buçgün et al., 2024). In contrast, FAB1 showed minimal group separation, possibly reflecting a ceiling effect at this level of task difficulty (Buçgün et al., 2024).

CDR-SOB showed more consistent group-differential longitudinal changes across all three groups, whereas MMSE did not clearly differentiate AD from MCI after controlling for baseline severity (Benoit et al., 2020; Sejunaite et al., 2024). This pattern aligns with prior reports suggesting that CDR-SOB may be more sensitive than MMSE for capturing longitudinal disease progression (Samtani et al., 2014; Williams et al., 2013), particularly in early and middle disease stages where MMSE exhibits well-documented ceiling and floor effects (Franco-Marina et al., 2010). In this context, the modest association between baseline FER and subsequent CDR-SOB, but not MMSE, suggesting that FER may capture aspects of clinical progression not fully reflected by global cognitive screening measures (Torres et al., 2015).

Notably, FER change rates showed a weak and non-significant correlation with MMSE change (*ρ* = 0.004, *p* = 0.960) but a modest, statistically significant correlation with CDR-SOB change (*ρ* = −0.195, *p* = 0.018). This dissociation suggests that FER trajectories are largely independent of global cognitive screening performance yet share modest overlap with changes in functional severity as indexed by CDR-SOB (Chaudhary et al., 2022). FER relies on the integrity of the amygdala, fusiform gyrus, and orbitofrontal–temporoparietal networks (Frank et al., 2019; Goodkind et al., 2012), which have been implicated in emotion processing and may be affected in AD-related neurodegeneration independent of hippocampal memory circuits (Chaudhary et al., 2022). The modest magnitude of the observed associations (*ρ* = −0.221 for baseline FER vs. CDR-SOB change rate) underscores that FER, as measured by the K-FAB, should not be interpreted as a standalone prognostic instrument at the current stage of evidence. Rather, these findings provide preliminary support for FER as a complementary—rather than replacement—marker in longitudinal AD monitoring, with the potential to capture aspects of functional vulnerability not reflected by global cognitive screening (Strijkert et al., 2022). Replication in larger, biomarker-confirmed, multi-center cohorts is required before clinical translation can be considered (Leuzy et al., 2025; Palmqvist et al., 2015).

Several limitations warrant acknowledgment. First, the follow-up duration (mean 1.59 years) was relatively short for a neurodegenerative study, and may have been insufficient to capture the full trajectory of FER decline, which appears to progress gradually based on cross-sectional data. Longer observational windows of three or more years will be needed to fully characterize longitudinal FER trajectories across the AD spectrum. Additionally, only 148 of 328 participants (45.1%) contributed longitudinal data, introducing the risk of attrition-related selection bias. Comparison of baseline characteristics between participants with and without follow-up data revealed that the longitudinal cohort was enriched for MCI and AD relative to SCD (MCI 45%, AD 34% vs. MCI 42%, AD 23%; *p* = 0.004), and had modestly lower baseline MMSE scores (23.5 vs. 24.5; *p* = 0.036), while baseline FER_mean did not differ between groups (*p* = 0.916). This pattern suggests that participants with milder cognitive concerns (SCD) were more likely to drop out, potentially attenuating observable FER differences at the less severe end of the spectrum. Second, the K-FAB employs static facial photographs, which may underestimate real-world facial emotion recognition ability compared to dynamic or naturalistic stimuli. Future studies should examine whether dynamic FER paradigms yield greater sensitivity to disease-related change across the AD spectrum. Third, biomarker confirmation (amyloid PET, CSF) was not uniformly available, precluding biologically confirmed AD classification and limiting disease-specific interpretation. All diagnoses were established using NIA-AA clinical criteria, and mixed or alternative pathologies cannot be excluded. Future studies should apply established AT(N) biomarker frameworks to determine whether FER-based predictions are specific to amyloid-tau pathology or reflect a broader neurodegenerative signal (Leuzy et al., 2025). Fourth, the absence of a cognitively healthy control group precludes direct comparison of FER trajectories against normative aging, making it difficult to determine what proportion of observed FER decline reflects disease-specific versus age-related change. Inclusion of healthy older adult controls is strongly recommended in future longitudinal designs. Finally, the observed associations were modest in magnitude (*ρ* = −0.221 for baseline FER vs. CDR-SOB change rate) and derived from exploratory analyses in a single-center cohort, warranting cautious interpretation. FER should not be considered a standalone prognostic instrument at this stage; rather, these findings support its potential as a complementary tool requiring replication in larger, biomarker-confirmed, multi-center cohorts before any clinical translation can be considered. Future studies incorporating neuroimaging, fluid biomarkers, and emerging digital approaches (Bergamasco et al., 2025; Jiang et al., 2024) to emotion analysis will be essential to elucidate the neural mechanisms underlying FER impairment and its relationship to AD pathophysiology.

## 5 Conclusion

Baseline facial emotion recognition was modestly associated with subsequent worsening of clinical severity, as measured by CDR-SOB, but not with changes in global cognitive screening performance (MMSE), in individuals spanning the pre-dementia to dementia continuum. FER change trajectories were largely independent of MMSE-measured cognitive change but modestly correlated with CDR-SOB–indexed functional decline (ρ = −0.195, *p* = 0.018), reflecting partially distinct yet overlapping aspects of disease-related change. CDR-SOB demonstrated more consistent sensitivity in detecting group-differential longitudinal change relative to MMSE. These findings suggest that FER assessment may serve a complementary—rather than standalone—role in longitudinal monitoring of individuals across the AD continuum. Given the modest magnitude of the observed associations and the exploratory nature of these analyses, clinical implementation is premature; replication in larger, biomarker-confirmed, multi-center cohorts remains an essential prerequisite.

## Data Availability

The data that support the findings of this study are not publicly available due to institutional regulations and ethical restrictions related to the protection of participant privacy. De-identified data may be made available upon reasonable request, subject to approval by the relevant institutional authorities. Requests for data access should be directed to the corresponding author (YS).
