# Supplementary material for: Baseline facial emotion recognition is associated with clinical decline across the Alzheimer’s disease spectrum: a multi-instrument longitudinal assessment study
Source: Front Aging Neurosci. 2026 Jun 30;18:1863175. doi: 10.3389/fnagi.2026.1863175 (PMC13364900; doi:10.3389/fnagi.2026.1863175)

**SUPPLEMENTARY FIGURE LEGENDS**

**Supplementary Figure 1.** Longitudinal Trajectories by Diagnostic Group (Follow-Up Cohort). Mean (±SE) scores for MMSE (left), CDR-SOB (middle), and FER Mean (right) at four time intervals (BL, ~1 yr, ~2 yr, >2 yr) in the follow-up cohort (n=148).

Annotations indicate sample size (n) at each time point.


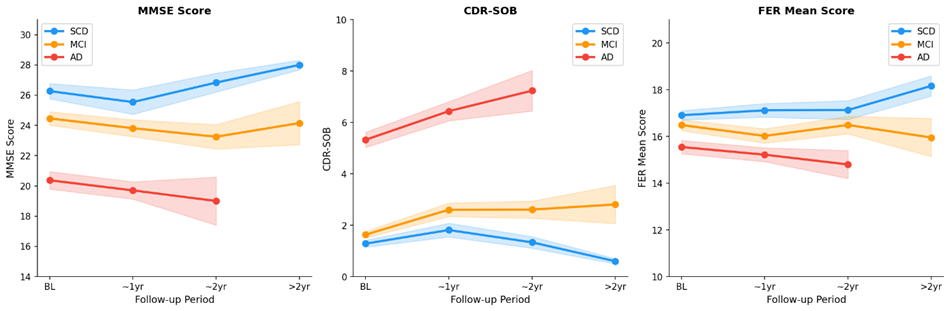


**Supplementary Figure 2.** Individual Trajectories (Spaghetti Plot) – Follow-Up Cohort. Individual-level longitudinal trajectories for MMSE (left), CDR-SOB (middle), and FER Mean (right) plotted against years from baseline. Bold lines represent group mean trajectories.


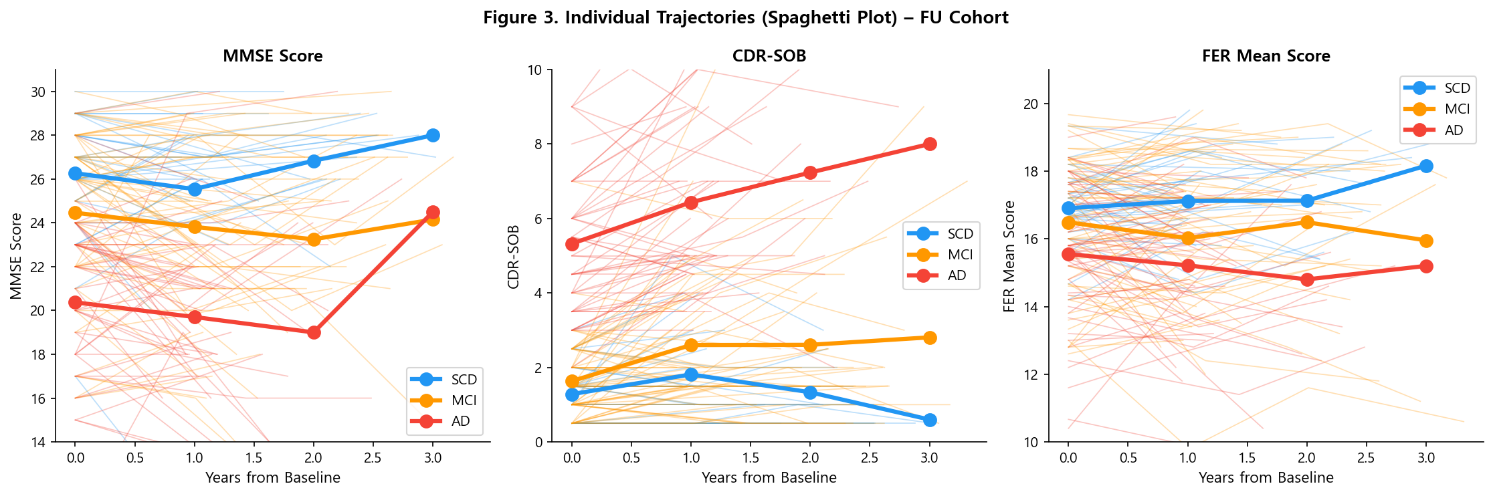

Supplement: Supplementary file 1 [file Data_Sheet_1.docx]
